# Experiments with Pressure Anaesthesia

**Published:** 1904-06

**Authors:** H. E. Sanders

**Affiliations:** Erin, Tenn.


### EXPERIMENTS WITH PRESSURE ANAESTHESIA.

By Dr. H. E. Sanders, Erin, Tenn.

The following is the history of a few cases in which I have used the pressure method of pulp extirpation as set forth in the October number, 1903, of Items of Interest by Dr. Clyde Davis. I commenced using the other pressure method several years ago and have used it to some extent, and with varying success up to the time I adopted the adrenalin and cocaine method which was about one month ago.

Lady about forty years old. Superior left lateral, disto-approximal cavity. Pulp not entirely exposed. Applied adrenalin and cocaine with pressure by unvulcanized rubber for about a minute. Cut in and applied again for about same time and removed pulp painlessly with not a drop of blood. Another case a day or so afterward was attended with so much pain that I desisted and applied arsenic, but I am now convinced that the trouble was in my not knowing how long to maintain the pressure. I only held it one minute whereas I believe, if I had held it three minutes, as the editoral in the November issue of ITEMS OF INTEREST directs, it would have worked as well as my other cases.

Girl about sixteen years old. Superior right central very large root. Pulp exposed. Applied cocaine and adrenalin for about one minute with pressure as before, and removed with very little pain and and some hemorrhage, which I stopped by means of cotton saturated in adrenalin and inserted in root. Had I applied pressure long enough I am convinced that there would have been absolutely no pain and no bleeding to amount to anything.

Lady about twenty-five years old. Superior right central. Applied cocaine and adrenalin for three minutes to cavity (pulp was not exposed). Cut down and removed painlessly and almost bloodlessly.

Same person as above. Superior left central. Pulp not quite exposed. Applied for three minutes; cut down and applied for three minutes more and remove painlessly and almost bloodlessly.

Same person as above. Pulp not quite exposed in superior right lateral. Applied for three minutes; cut down and applied for three minutes more; cut down again and applied a third time for three minutes, when I removed absolutely without any pain and very little bleeding.

Man about thirty-five, fireman on passenger train, came to my office at night suffering from the most violent toothache I have ever seen, in a lower right third molar. He had put something in to stop the pain and in pressing it in had pressed part of the thin bottom of the cavity into the pulp. I removed this softened dentine, applied cocaine and adrenalin with pressure for three minutes and removed pulp without pain.

Negro boy about twenty years old; superior right first molar; applied for three minutes to cavity. Pulp was not quite exposed. Removed it without pain and no bleeding.

In all these cases, except VI. and VII. the roots were filled immediately and the teeth filled with gold. The others were left because of a lack of time to finish the operation. In none of them was there any soreness to amount to anything. What little was present in one case was relieved by an application of T. Aconite and T. Iodine equal parts. In case VI., I know of nothing that would have given this man immediate relief except possibly extraction, or perhaps morphine might have relieved him in the course of an hour. He was suffering intensely, muscles were twitching and great beads of perspiration rolling off his face; yet in four minutes from the time he entered my office he was perfectly easy.

I shall always be grateful to the gentleman who has given us this method.

---

